# Effects of Atmospheric and Room Temperature Plasma (ARTP) Mutagenesis on Physicochemical Characteristics and Immune Activity In Vitro of *Hericium erinaceus* Polysaccharides

**DOI:** 10.3390/molecules24020262

**Published:** 2019-01-11

**Authors:** Lingli Zhu, Di Wu, Henan Zhang, Qiaozhen Li, Zhong Zhang, Yanfang Liu, Shuai Zhou, Wenhan Wang, Zhengpeng Li, Yan Yang

**Affiliations:** 1 Institute of Edible Fungi, Shanghai Academy of Agricultural Sciences, Key Laboratory of Edible Fungi Resources and Utilization (South), Ministry of Agriculture, National Engineering Research Center of Edible Fungi, Shanghai 201403, China; 13262587402@163.com (L.Z.); wudi@saas.sh.cn (D.W.); henanhaoyun@126.com (H.Z.); liqiaozhen-345@163.com (Q.L.); aliu-1980@163.com (Y.L.); simonzsz@gmail.com (S.Z.); wangwenhan@saas.sh.cn (W.W.); lizp_ln@126.com (Z.L.); 2 College of Food Science & Engineering, Shanghai Ocean University, Shanghai 201306, China

**Keywords:** *Hericium erinaceus*, polysaccharide, atmospheric pressure room temperature plasma, physicochemical properties, biological activities

## Abstract

The polysaccharide is the main active substance contained in *Hericium erinaceus* and is commonly used in the treatment of neurasthenia, tumors, and digestive diseases. Six intracellular polysaccharide components were obtained from *H. erinaceus* fruiting bodies cultivated by ARTP (atmospheric and room temperature plasma) mutagenic strain (321) and the original strain (0605), respectively. This study was designed to investigate the physicochemical characteristics of these polysaccharide components and their potential immunomodulatory activities on RAW264.7 macrophages. The results showed that the yield of fruiting body cultivated by mutated strain increased by 22% and the polysaccharide content improved by 16% compared with the original one owing to ARTP mutagenesis. The molecular weight distribution and the monosaccharide compositions of polysaccharide components from *H. erinaceus* induced by ARTP mutagenesis were significantly different from that of the original one. The NO, IL-6, IL-10, IL-1β, and TNF-α production activities of macrophages were enhanced by stimulation of 20% ethanol precipitated polysaccharides from *H. erinaceus* induced by ARTP mutagenesis. These results indicated that ARTP is an efficient and practical method for high polysaccharide content breeding of the *H. erinaceus* strain and this provided a reference for obtaining high quality resources and healthy product development from *H. erinaceus*.

## 1. Introduction

*Hericium erinaceus* (*H. erinaceus*) is commonly consumed as edible or medicinal sources in China and other oriental countries and is also known as lion’s mane mushroom or hedgehog mushroom [1,2]. Previous studies indicated *H. erinaceus* has great medicinal value due to its various beneficial effects, especially for gastrotherapy [3,4]. To date, many bioactive compounds have been isolated and identified from *H. erinaceus*, such as hericenones, erinacines, glycoprotein, polysaccharides, steroids, alkaloids, and hericins [5,6,7]. Among these compounds, polysaccharides isolated from its fruiting bodies are supposed to be one of the major bioactive compounds, which possess various pharmacological activities, such as immuno-modulating, anti-tumor, antioxidant, gastro-protective, wound healing, and anti-mutation activities [8]. In recent years, a number of functional foods and medicines made from *H. erinaceus* fruiting bodies have appeared on the Chinese market, which contain polysaccharides as the main functional ingredient [9].

High-yielding fruiting body production is the traditional breeding goal to get more economic benefits from cultivation. For functional purposes, strains with highly desired metabolites, such as polysaccharides, are promising to be able to harvest high-quality fruiting bodies for health food development [10]. Strain improvement for increasing biological yield and the polysaccharide content is both needed. Some strain-breeding methods such as ion beam tries, ultraviolet radiation, ^60^Co-γ irradiation combined with ultraviolet and X-ray mutagenesis breeding methods has been used to improve the polysaccharide content of *H. erinaceus* [11,12,13]. However, changes in the structural properties and activities of polysaccharides improved by mutagenesis has not been reported. Atmospheric pressure room temperature plasma (ARTP) is a novel and effective physical mutagenesis technology for microbial mutation breeding and creating a mutant library of microorganisms [14,15]. ARTP can be generated at atmospheric pressure by radio-frequency power and the plasma can be controlled at room temperature, which is beneficial for microbial DNA mutation and altering the metabolic networks of the target microbes [16]. ARTP technology has already been applied in many microbial breeding studies. Ren et al. obtained higher acarbose producing strains, which used the method of screening the strains for susceptibility to penicillin after treatment with ARTP [17,18]. Furthermore, He et al. used ARTP to construct a library of mutant strains for excellent strain screening, and obtained an excellent mutant strain which was resistant to low temperature and fast growing [19]. Another study used a new method to generate mutations in *Blakeslea trispora* to improve the fermentation efficiency of lycopene, which is a plasma jet driven by an active helium atom supplied with an ARTP biological breeding system [20]. In our previous study, ARTP was first used as induced mutation tool to irradiate protoplasts of *H. erinaceus* and a new strain was obtained with higher polysaccharide production, and the polysaccharide content in liquid fermentation mycelium and fruiting bodies were both improved. However, whether there were any changes of physicochemical characteristics and activities of polysaccharides from the *H. erinaceus* mutant strain are unclear.

For a living organism, immunity is delimited as the ability of immune recognition and destruction of external harmful substances, which plays a significant role in our health [21,22]. The immunological regulation mechanisms of polysaccharides are promoting immune allelotaxy, enhancing macrophage phagocytosis function, advancing lymphocyte proliferation, increasing humoral immune function, upgrading immune cytokine and its mRNA expression, and so on [23,24,25]. Macrophages are a type of important immune cells and the body’s first line of defense against infection [26,27], and can neutralize foreign substances, cancer cells, and infectious microbes through phagocytosis, chemotaxis, surveillance, and by releasing proinflammatory cytokines including nitric oxide (NO) [28,29,30,31]. Therefore, macrophages have been widely used to judge the immune activity of polysaccharides in vitro by manifold parameters analysis [32,33,34]. The production of NO, TNF-α, IL-6, IL-10, and IL-1β is an important part of the immune response to many inflammatory stimuli.

Based on previous studies, the aim of this study was to investigate the differences of physicochemical characteristics and immune activity between polysaccharides isolated from fruiting bodies cultivated by ARTP mutagenic strains (321) and the original strain (0605) of *H. erinaceus*. Moreover, macrophage stimulation and cytokine production assays in vitro were tested to assess the effect of ARTP mutagenesis on polysaccharide immunostimulant activities.

## 2. Results and Discussion

### 2.1. Comparative Analysis of Agronomic Characters and Polysaccharide Content

**Figure 1:**
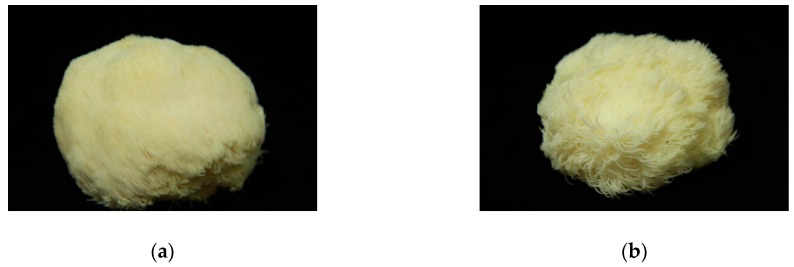
The fruiting bodies of *H. erinaceus* 321 (**a**) and *H. erinaceus* 0605 (**b**).

The mutagenic strain 321, which screened from hundreds of strains bred through ARTP, had been identified by an antagonism test and random amplification polymorphic DNA (RAPD) analysis to be a new strain with changes in genetic material with the parent strain (results will soon be published in another journal). After five generations of culture, the mutant strain 321 showed excellent genetic stability and morphological stability. The mutant strain 321 and original strain 0605 of *H. erinaceus* were cultivated under industrialized conditions and agronomic characters of the fruiting bodies, harvested at mature stage, were detected. The fruiting bodies of strain 321 are bigger than that of 0605 in external forms, as shown in Figure 1, and the fleshy quality of the 321 fruiting body is tighter than that of 0605, as shown in Table 1. Furthermore, compared to the spiny length of 0605, the spiny length of 321 is longer and the individual yield of the 321 fruiting body is higher than that of 0605. Owing to ARTP mutagenesis, the yield of fruiting bodies increased by 22% and the polysaccharide content of the mutated strain improved by 16% compared with the original one, as shown in Table 1. Results also indicated there was a positive correlation between the yield of fruiting body and the content of polysaccharides. Normally, the polysaccharide content in the fruiting body is not only correlated with the fruiting body growing stage, but also with the cultivating strain of the fruiting body [35]. Zhou et al. studied the yield and polysaccharide content of fruiting bodies cultivated from eight different *Ganoderma lucidum* strains and found that there were significant differences in the yield and polysaccharide content among the fruiting bodies of the eight strains, and the polysaccharide content of the strains with high yield was not necessarily high [36]. The results provide theoretic background for further quality improvement of *H. erinaceus* in cultivation. 

### 2.2. Effect of ARTP on Polysaccharide Molecular Weight Distribution Pattern

**Figure 2:**
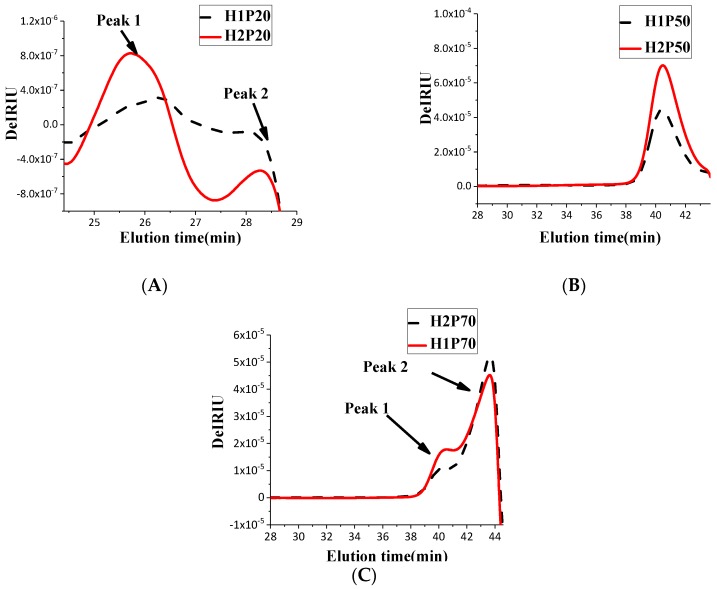
HPSEC-MALLS-RI (high performance size exclusion chromatography equipped with multiple angle laser light scattering and refractive index) chromatograms of 20%, 50%, and 70% ethanol precipitated polysaccharides from *H. erinaceus*. (**A**) HPSEC-MALLS-RI chromatograms of 20% ethanol precipitated polysaccharides; (**B**) HPSEC-MALLS-RI chromatograms of 50% ethanol precipitated polysaccharides; (**C**) HPSEC-MALLS-RI chromatograms of 70% ethanol precipitated polysaccharides; H1P and H2P represent the original and atmospheric and room temperature plasma (ARTP) mutant strain, respectively.

The molecular weight distribution pattern of 20%, 50%, and 70% ethanol precipitated polysaccharide fractions of original strain 0605 (H1P20, H1P50, H1P70) and the ARTP mutant strain 321 (H2P20, H2P50, H2P70) were analyzed by high performance size exclusion chromatography equipped with multiple angle laser light scattering and refractive index detectors (HPSEC-MALLS-RI), as shown in Figure 2. The molecular weights and percentages of each polysaccharide fraction were calculated by ASTRA data analysis software and the data are summarized in Table 2. The results showed that the molecular weight distribution of 20% ethanol precipitated polysaccharides of *H. erinaceus* induced by ARTP mutagenesis was significantly different from that of the original one, as shown in Figure 2A, while the differences of molecular weight distribution of the 50% and 70% ethanol precipitated polysaccharides between native and mutated strains were negligible, as shown in Figure 2B,C.

As Figure 2A shows, 20% alcohol precipitated polysaccharide fraction H1P20 has two peaks, of which the molecular weight of Peak1 was about 6300 kDa, the area occupied 51.7%, and that of Peak2 was about 230 kDa, accounting for 48.3%, as shown in Table 2; however, the molecular weight of Peak1 of H2P20 isolated from the mutant strain was about 21,000 kDa, accounting for 78.8%. The results showed that the distribution of molecular weight of the fruiting body polysaccharide was changed by ARTP mutagenesis. The molecular weight of polysaccharides which were above 10 million increased by ARTP mutagenesis, while the proportion of molecular weight of polysaccharides below 100 kDa was similar. The biological activity of polysaccharides is closely related with their molecular weight, meanwhile, senior conformation of polysaccharides with large molecular weight is also related to their immune activity in vitro [37]. Whether the changed molecular weight distribution by ARTP mutation will affect the biological activity of polysaccharides needs to be further explored. ARTP mutagenesis can damage the genome diversity and produce large-scale gene mutations [38], which will be the factor for the increase of macromolecular polysaccharide components and the change of the polysaccharide fraction proportion of *H. erinaceus*. The results provide some ideas for directional breeding of high polysaccharide production.

### 2.3. Effect of ARTP on Monosaccharide Composition of Hydrolyzed Polysaccharide Fractions

The monosaccharide composition and molar ratio of six polysaccharide fractions obtained from the fruiting bodies of original and ARTP mutant strains were determined by high performance anion chromatography (HPAEC). The monosaccharide composition of polysaccharide fractions from ARTP mutagenesis was significantly different from that of the original one, as shown in Table 3.

The 20% ethanol precipitated polysaccharides were mainly composed of fucose, arabinose, galactose, glucose, and mannose. Among them, the proportion of galactose in the polysaccharide component of H2P20 was higher than that of H1P20, and the proportion of arabinose in the polysaccharide component of H2P20 was significantly lower than that of H1P20, as shown in Table 3. Fifty per cent alcohol precipitated polysaccharides were mainly composed of fucose, galactose, glucose, and mannose. Among them, the proportion of fucose in the polysaccharide component of H2P50 was about six times that of H1P50. Meanwhile, the proportion of galactose and glucose in the polysaccharide component of H2P50 was about two times that of H1P50, as shown in Table 3. Among 70% ethanol precipitated polysaccharide fractions, the proportion of fucose in the polysaccharide H2P70 was about five times that of H1P70 and the proportion of galactose of H2P50 was about three times that of H1P70, as shown in Table 3. The results showed that the monosaccharide proportion in the polysaccharide component of H2P50 and H2P70 changed greatly compared to that of H1P50 and H1P70. These illustrated that the structural characteristics of polysaccharides also changed after ARTP mutation, and whether the structural changes affect the activity of polysaccharides needs further study.

### 2.4. Fourier Transform Infrared Spectrum Analysis

**Figure 3:**
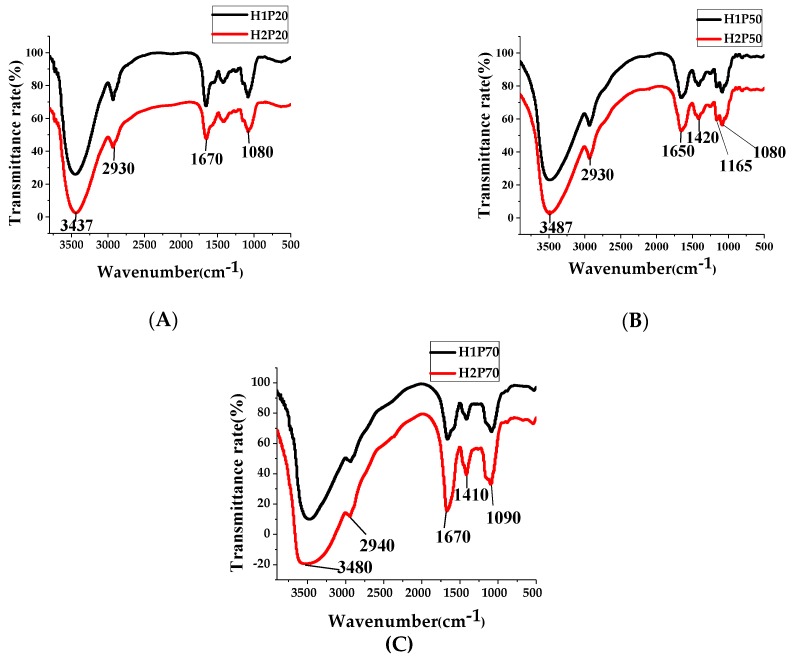
FT-IR spectrum of 20%, 50%, and 70% ethanol precipitated polysaccharides from fruiting bodies of *H. erinaceus.* (**A**) IR spectrum of 20% ethanol precipitated polysaccharides; (**B**) IR spectrum of 50% ethanol precipitated polysaccharides; (**C**) IR spectrum of 70% ethanol precipitated polysaccharides; H1P20 (H1P50, H1P70): 20% (50%, 70%) ethanol precipitated polysaccharides from the original *H. erinaceus*; H2P20 (H2P50, H2P70): 20% (50%, 70%) ethanol precipitated polysaccharides from the ARTP mutagenic *H. erinaceus*.

Fourier transform infrared (FT-IR) spectrums of six polysaccharide fractions from original and ARTP mutant fruiting bodies of *H. erinaceus* are shown in Figure 3. Absorption bands at 3437 and 2930 cm^−1^ were attributed to the O-H stretching vibration and the C-H stretching vibration, respectively, as shown in Figure 3A, and these two peaks are the typical absorption peaks of carbohydrates. The absorption band at 1670 cm^−1^ was due to the C=O stretching vibration. The absorption band at 1080 cm^−1^ was the typical absorption peak of pyranose and this indicated that the polysaccharide component was composed of a glucoside bond and another one. The spectrum of H2P20 was similar to that of H1P20, from which could be concluded that ARTP mutagenesis did not change the basic structure of macromolecular polysaccharides. The FT-IR spectrum of H1P50 and H2P50 are shown in Figure 3B, and the spectrum was similar to that of H1P20 and H2P20 with little difference on absorption bands. However, the absorption band at 1090 cm^−1^ in the spectrum of H2P70 increased a little compared with H1P70, as shown in Figure 3C), which was consistent with a significant increase in the glucose composition of H2P70. This indicated ARTP mutagenesis increased the type of glucoside bond, which may be related to the enhanced expression of glucosidase. The specific differences in the structural characteristics of polysaccharides between H1P and H2P still need further study.

### 2.5. Effect of ARTP on Nitric Oxide Production from Macrophages

#### 2.5.1. Cell Viability Assay

**Figure 4:**
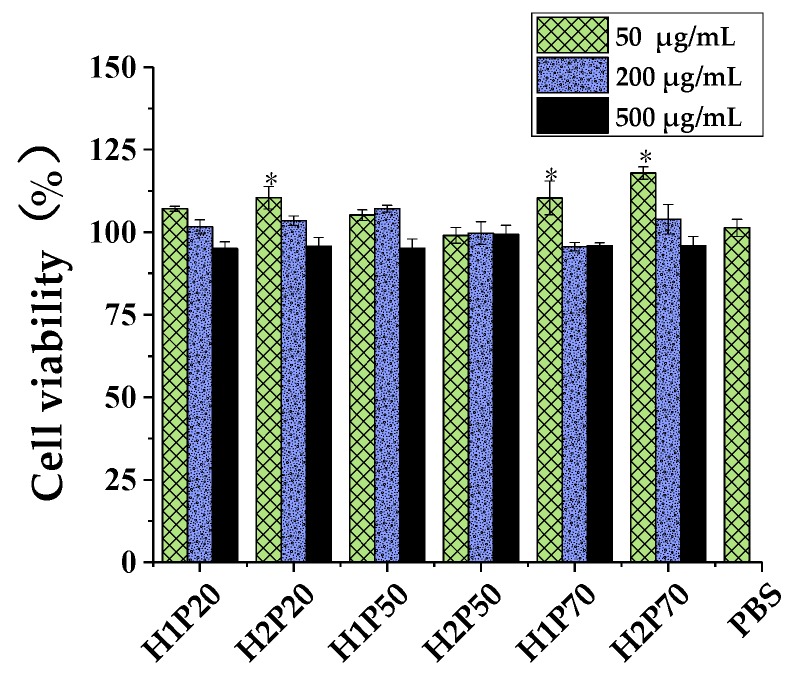
Viability of macrophage cells. Each value represents the mean ± SD. * *p* < 0.05 compared to the negative control (phosphate buffered saline (PBS) treatment).

The determination of cell viability plays an important role in toxicity tests. It is a basic tool for screening new drugs and chemicals and provides preliminary data prior to performing in vivo and clinical studies on account of various kinds of functions, such as mitochondrial enzyme activity, cell membrane permeability, ATP production, and cellular uptake activity [39]. Alamar blue assays have been widely used in cell viability and cytotoxicity tests [40]. As shown in Figure 4, the cell viability rates of fractions treatment were all above 95% at the concentration from 50 to 500 µg/mL; the decrease of cell viability was within acceptable limits. The results showed the mutated *H. erinaceus* would be safe.

#### 2.5.2. Nitric Oxide Released by RAW264.7 Macrophages

**Figure 5:**
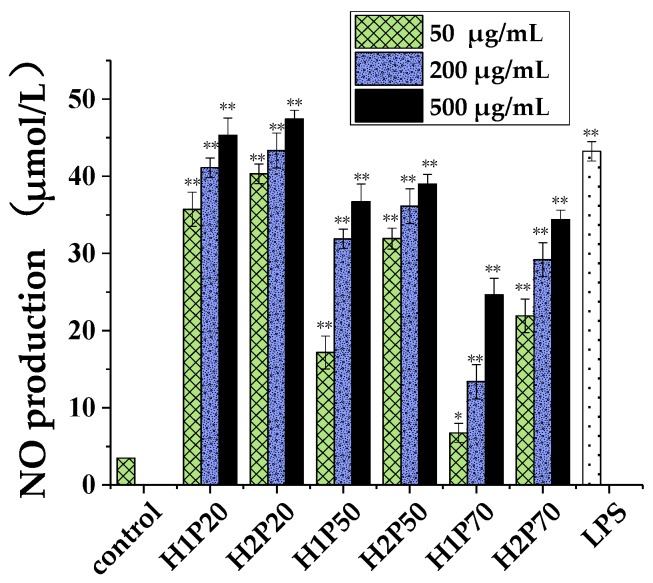
Effects of polysaccharides extracted from the fruiting bodies of *H. erinaceus* on NO production from RAW264.7 macrophage cells. Control: phosphate buffer; LPS: lipopolysaccharide, positive control (1 μg/mL). H1P20 (H1P50, H1P70): 20% (50%, 70%) ethanol precipitated polysaccharides from the original *H. erinaceus*; H2P20 (H2P50, H2P70): 20% (50%, 70%) ethanol precipitated polysaccharides from the ARTP mutagenic *H. erinaceus*. Each value represents the mean ± SD. * *p* < 0.05, ** *p* < 0.01 compared to the negative control (PBS treatment).

Enhancing macrophage immune responses is one of the immunological regulation mechanisms of polysaccharides, and generally used to evaluate the in vitro immune activities of polysaccharide fractions [41]. Herein, six polysaccharide fractions extracted from original and ARTP mutant fruiting bodies of *H. erinaceus* were used to compare the macrophage immune activities by determining the NO production of RAW264.7 cells. As shown in Figure 5, the NO concentration of the culture supernatant of the RAW264.7 cells were significantly increased by treatment with different doses of 20%, 50%, and 70% ethanol precipitated polysaccharides from both original and ARTP mutant fruiting bodies of *H. erinaceus* (50, 200, and 500 μg/mL) in a concentration dependent manner. Compared to 50% and 70% ethanol precipitated polysaccharides, 20% ethanol precipitated polysaccharides significantly increased higher NO secretion. At the same time, H1P20 and H2P20 showed higher macrophage activation activity at the low concentration of 50 μg/mL than other groups, including lipopolysaccharide (LPS). The macrophage activation activity of 20% ethanol precipitated polysaccharides with a large molecular weight was better than that of 50% and 70% ethanol precipitated polysaccharides, indicating that macromolecular polysaccharides showed better in vitro immune activity.

The NO production of the RAW264.7 cells stimulated by H2P20 maintained a similar but a little increased level with that of H1P20, which inferred that the macrophage activation of 20% ethanol precipitated polysaccharides was changed slightly by the ARTP mutagenesis, as shown in Figure 5). Otherwise, the NO production of the RAW264.7 cells stimulated by H2P50 was significantly increased at the low concentration of 50 μg/mL than that of H1P50. The NO production of the RAW264.7 cells stimulated by H2P70 was significantly increased at 50, 200, and 500 μg/mL than that of H1P70, which indicated that ARTP mutagenesis enhanced the macrophage activation activity of polysaccharides with molecular weights between 10–100 kDa and the activity of polysaccharides may be relation to the molecular weight and structure of the sugar chain. Some reports showed that the high molar mass was thought to be favorable structural parameters for their immunological and anti-tumor activity [42,43,44,45]. In this study, the polysaccharide fraction with a molecular weight of 5000–20,000 kDa also showed higher immunological activity at the concentration from 50 to 500 μg/mL. The results further indicated that the molecular weight, monosaccharide composition, and other structural characteristics of *H. erinaceus* polysaccharides were related to their immune activity in vitro.

### 2.6. Effect of ARTP on Immunostimulatory Activity

**Figure 6:**
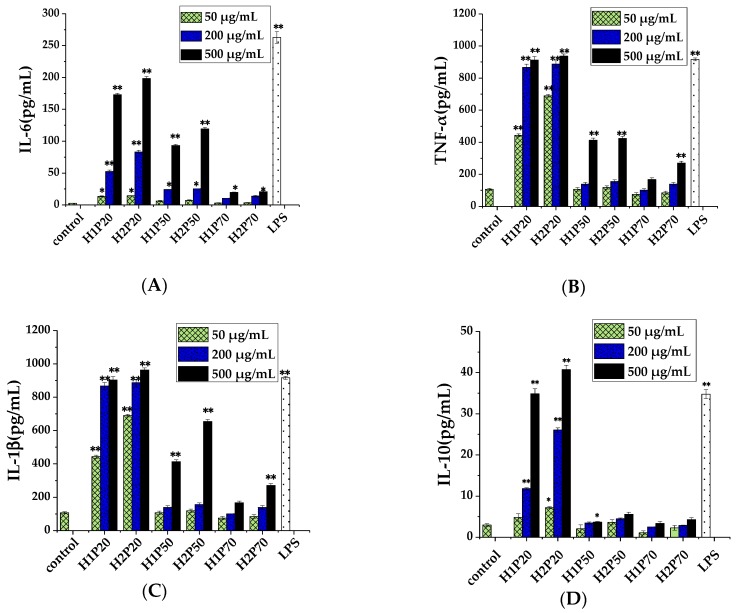
Effect of 20% (50%, 70%) ethanol precipitated polysaccharides on IL-6 (**A**), TNF-α (**B**), IL-1β (**C**), and IL-10 (**D**) from THP-1 cells. H1P20 (H1P50, H1P70): 20% (50%, 70%) ethanol precipitated polysaccharides from the original *H. erinaceus*; H2P20 (H2P50, H2P70): 20% (50%, 70%) ethanol precipitated polysaccharides from the ARTP mutagenic *H. erinaceus*. Each value represents the mean ± SD. * *p* < 0.05, ** *p* < 0.01 compared to the negative control (PBS treatment).

IL-6 and TNF-α are important innate proinflammatory cytokines involved in host defense, inflammation, and apoptosis. IL-1β is an antigen-presenting cell proinflammatory cytokine in the upstream of the immune response and it can stimulate the production of a variety of inflammatory mediators [46]. IL-10 is a key anti-inflammatory cytokine that plays a critical role in the control of the immune response. It is believed that the increased IL-10 levels during inflammation may counteract the inflammatory process in order to reestablish homeostasis [47]. By ELISA analysis, cellular release of IL-6, TNF-α, IL-1β, and IL-10 were shown to be elevated by H1P and H2P in a significant dosage dependent manner, as shown in Figure 6.

As shown in Figure 6A, the effect of H1P and H2P on the production of IL-6 was determined in the culture supernatants of macrophages after 48 h of cultivation. Compared to H1P70 and H2P70 groups, H1P20, H2P20, and H1P50, H2P50 significantly increased IL-6 production at 500 μg/mL. IL-6 production stimulated by H2P20 (500 μg/mL and 200 μg/mL) groups were higher than that of H1P20 groups. At the same time, H1P20 obviously increased TNF-α production more than the H1P50 and H1P70 groups at 50, 200, and 500 μg/mL, respectively, and the H2P20 group also significantly increased TNF-α production compared with the H2P50 and H2P70 groups, as shown in Figure 6B. The H2P20 group had higher stimulating activity on TNF-α production at 50 μg/mL than that of H1P20 group, as shown in Figure 6B. These demonstrated that macromolecular polysaccharide fractions had higher stimulating activity on IL-6 and TNF-α production, and polysaccharide fractions from the fruiting bodies cultivated by ARTP mutagenic strain had higher stimulating activity on IL-6 and TNF-α production than that from the original one. Meanwhile, H1P20 obviously increased IL-1β production more than the H1P50 and H1P70 groups at 50, 200, and 500 μg/mL concentrations, respectively, and the H2P20 group also significantly increased IL-1β production compared with the H2P50 and H2P70 groups, as shown in Figure 6C. The H2P20 group had higher stimulating activity on IL-1β production at 50 μg/mL than that of the H1P20 group, as shown in Figure 6C. The H2P20 groups obviously increased IL-10 production at 200 and 500 μg/mL, respectively, compared with the H2P50 and H2P70 groups which had little effect on IL-10 production, as shown in Figure 6D. The H2P20 (200 μg/mL) group had higher stimulating activity on IL-10 production than that of the H1P20 group, as shown in Figure 6D. Cytokines are produced by immune and non-immune cells when challenged by various environmental or inflammatory insults and mediate almost all phases of the inflammatory process. It is believed that increased expression of IL-6, TNF-α, IL-1β, and IL-10 lead to increased immunological activity [46,47]. These results suggested that macromolecular polysaccharide fractions from the fruiting bodies of *H. erinaceus* were important for its immunological activity, and the polysaccharide fractions with large molecular weight distribution from the ARTP mutagenic *H. erinaceus* improved proinflammatory cytokine production. This indicated that the immunological activity of polysaccharides extracted from *H. erinaceus* can be enhanced by strain mutagenesis.

## 3. Materials and Methods

### 3.1. Materials and Chemicals

*H. erinaceus* strain 0605 was obtained from the Herbarium of Edible Fungi Culture Collection Center Branch of the China Culture Collection of Agricultural Microorganisms (Shanghai, China).

*H. erinaceus* mutant strain 321 was bred through the ARTP method from parental strain 0605 according to the literature with minor modifications [19]. First, protoplasts of strain 0605 were prepared and used for ARTP mutation. In the present mutation, pure helium was used as the plasma working gas, 20 μL protoplast suspensions of *H. erinaceus* (1 × 10^7^ cells/mL), suspended in 0.6 mol/L mannitol stable seepage (with equal volume 10% glycerin as a protective agent), were placed on a stainless-steel minidisc. The operating parameters of the ARTP system (Yuan Qing Tian Mu Biotechnology Co., Ltd., Wuxi, China) were as follows: radio-frequency (RF) power input of 120 W, treating distance of 2 mm, the helium gas flow rate of 8 SLM (standard liter per minute), treating time of 30 s. After being treated by ARTP, the *H. erinaceus* protoplast suspensions were transferred onto a mutant screening plate and cultured at 26 °C in the dark for 5 days to screen regenerated colonies and calculate the lethality rate and positive mutation rate. After genetic stability, morphological stability, and RAPD analysis, among the hundreds of mutant strains, 321 mutant strains exhibited significantly enhanced polysaccharide yield compared with the original strain.

The human monocytic cell line THP-1 was purchased from the Type Culture Collection of the Chinese Academy of Sciences (Shanghai, China).

RAW264.7 cells, a murine macrophage cell line, was purchased from the Type Culture Collection of the Chinese Academy of Sciences (Shanghai, China).

### 3.2. Fruit Body Cultivation

*H. erinaceus* strain 0605 and 321 were cultivated in polypropylene bags filled with solid medium consisting of: 30% (*w*/*w*) sawdust, corncob 40% (*w*/*w*), cottonseed shell 15% (*w*/*w*), corn flour 2% (*w*/*w*), wheat bran 6% (*w*/*w*), rice bran 5% (*w*/*w*), 1% (*w*/*w*) gypsum, lime 1% (*w*/*w*), respectively. After inoculation with fungal mycelium, the bags were kept in the dark at 25 °C and 70% relative humidity for 25 days, and were then transferred to a ventilated field at 16 °C and 90% relative humidity for 12 days for growing fruiting bodies, and then was harvested at its mature stage.

### 3.3. Extraction and Isolation of Polysaccharide Components

**Figure 7:**
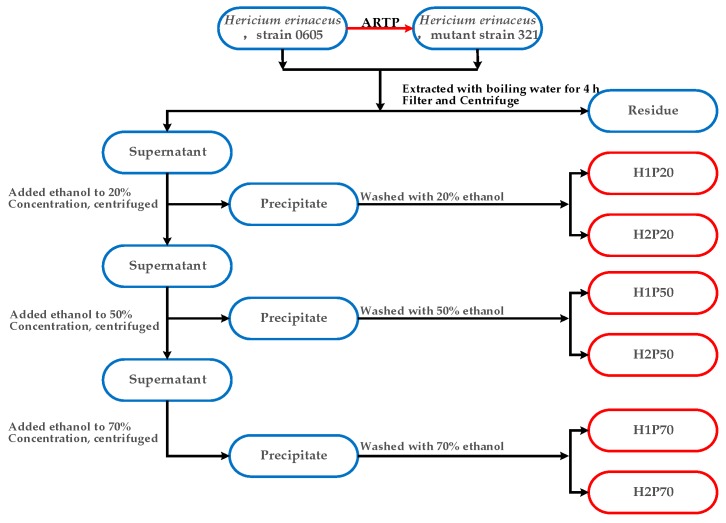
Stepwise extraction and isolation procedure of polysaccharides from fruiting bodies cultivated by two *H. erinaceus* strains.

The fruiting bodies of 0605 and 321 harvested at mature stage were dried at 55 °C for 48 h and then grounded into small pieces for polysaccharides extraction. Samples (1000 g) were extracted three times with 18 L of distilled water at 100 °C for 4 h, respectively. Combined filtrates were concentrated 3-fold under reduced pressure, then absolute EtOH was added to reach the concentration of 20% (*v/v*), and the precipitate was collected by centrifugation (Beckman Coulter, Inc, Brea, CA, USA) (9000× *g*, 30 min). After being washed three times with 20% EtOH, precipitates were dissolved in distilled water and dialyzed (cut-off 3500 Da) against water for three days at 4 °C with the following freeze-dry to obtain two fractions, named as H1P20 and H2P20, which were isolated from the fruiting body of 0605 and 321, respectively. Meanwhile, absolute EtOH was added to each of centrifuged supernatants to a final concentration of 50% and the precipitates (H1P50 and H2P50) were collected by centrifugation as above. Finally, the EtOH concentration in the supernatants was increased to 70% and the collected precipitates designated H1P70 and H2P70, respectively. The protocols of extraction and the isolation process of six polysaccharide components from two different strains are shown in Figure 7.

### 3.4. Total Polysaccharide Content

Total polysaccharide content of six polysaccharide components were determined spectrophotometrically (BIO-TEK, Winooski, VT, USA) at 490 nm using the phenol-sulfuric acid method with d-glucose as the standard [41].

### 3.5. Fourier Transform Infrared Spectrum Analysis of Polysaccharide Components

Infrared spectra of the six polysaccharide components were recorded with a Fourier transform infrared FT-IR spectrometer (Thermo Fisher Scientific, Waltham, MA, USA) in the range 4000–400 cm^−1^ using the KBr disk method [48].

### 3.6. Molecular Weight Distribution among the Polysaccharide Components

Six polysaccharide fractions (5 mg) were dissolved in 1 mL of phosphate buffer solution (0.15 M NaNO_3_ and 0.05 M NaH_2_PO_4_ containing 0.02% (*w/w*) NaN_3_, pH = 7) and centrifuged at 13,000× *g* for 30 min and the supernatant was passed through a 0.22 µm filter for analysis. Molecular weight distribution patterns among the six polysaccharides components were determined by high-performance size exclusion chromatography (HPSEC) equipped with multiple detectors: a refractive index detector (RI) and a UV detector (Waters, Milford, MA, USA) for assessing concentration, a multiple angle laser light scattering detector (MALLS, Wyatt Technology, Santa Barbara, CA, USA) for direct molecular determination. Chromatographic analysis column selected TSK PWXL6000 (7.8 × 300 nm) (Tosoh, Toyosawa, Fukuroi, Shizuoka, Japan) gel filtration column linked a TSK PWXL4000 (7.8 × 300 nm) gel filtration, which were eluted with phosphate buffer at a flow rate of 0.5 mL/min, the temperature of the columns and RI detector (Waters, Milford, MA, USA) were constant at 35 °C, and the wavelength of laser detector was 623.8 nm. The refractive index increment (dn/dc) of polysaccharide in solution was set to 0.146 mL/g. Data acquisition and analysis were carried out using ASTRA software (Version 6.1.1, Wyatt Technology, Santa Barbara, CA, USA).

### 3.7. Monosaccharide Composition Analysis

Samples (2 mg) of six polysaccharide components were hydrolyzed with 3 mL 2 M trifluoroacetic acid (TFA) at 110 °C for 4 h. After hydrolysis, hydrolysates were dried with a Termovap sample concentrator, and then 3 mL methanol was added and dried repeatedly three times until the TFA was completely removed. The monosaccharide compositions were determined by a high-performance anion exchange chromatography (HPAEC) system (Dionex ICS-2500, Dionex, Sunnyvale, CA, USA) equipped with a CarboPac™ PA20 column (3 mm × 150 mm, Dionex, USA) and a pulsed amperometric detector (Dionex, USA). The column was eluted with 2 mM NaOH (0.45 mL/min) followed by 0.05 to 0.2 M NaAc at 30 °C. The monosaccharide compositions and content of polysaccharide components were determined using d-Gal, d-Glc, d-Ara, l-Fuc, l-Rha, d-Man, d-Xyl, d-Fru, d-Rib, d-GluA, and d-GalA (Sigma-Aldrich, St. Louis, MO, USA) as the standards.

### 3.8. RAW264.7 Macrophages Trial

#### 3.8.1. Cell Viability Assay

RAW264.7 macrophages were plated in a cell culture dish, and cells were allowed to adhere and grow for approximately 48 h at 37 °C, 5% CO_2_ before proceeding with the assay. Cells were counted in a Z Series Counter (Beckman Coulter, Brea, CA, USA) and adjusted to 2 × 10^5^ cells/mL. The cell suspension (180 µL) was added to the 96-well plate with 20 µL different concentrations of polysaccharide fractions per pore. Phosphate buffered saline (PBS) was served as the negative control. After incubation for 48 h at 37 °C, 5% CO_2_, alamar blue reagents (20 µL) were added to each well, and then cultured for another 4 h. The absorbances at 570 nm and 600 nm were determined by an ELISA microplate reader. The cell viability rate was calculated according to protocol.

#### 3.8.2. Nitric Oxide Released by RAW264.7 Macrophages

Polysaccharide components were dissolved in a PBS solution at various concentrations (final concentration of 50, 200, and 500 μg/mL). RAW 264.7 cells were cultured in Dulbecco’s modified Eagle medium, containing 10% fetal bovine units/mL penicillin A, and 100 U/mL streptomycin (Amersco Co., Solon, OH, USA) at 37 °C in a 5% carbon dioxide (CO_2_) atmosphere. Aliquots of RAW264.7 cell suspension (180 µL; 5 × 10^5^ cells/mL) and 20 µL of test samples were added to each well of a 96-well plate and incubated at 37 °C in a 5% CO_2_ atmosphere for 48 h [49,50]. Supernatants (100 μL) were then collected and mixed with 50 μL Griess reagent [1% (*w*/*v*) sulfanilamide, 0.1% (*w*/*v*) naphthyl ethylenediamine dihydrochloride, 2% (*v*/*v*) phosphoric acid] and incubated at room temperature for 10 min. Lipopolysaccharide (LPS, 1 µL/mL) and phosphate buffered saline (PBS) served as the positive and negative controls, respectively. Nitric oxide production was carried out by comparing the absorbance at 543 nm against a standard curve generated using a series of concentrations of NaNO_2_.

### 3.9. THP-1 Macrophage Differentiation Trial

#### 3.9.1. Cell Culture

Cells were cultured in Roswell Park Memorial Institute medium (RPMI-1640 medium) added on 100 IU/mL penicillin and 100 μg/mL streptomycin, 1% sodium pyruvate, 0.5% β-mercaptoethanol (Sigma-Aldrich, USA), and 10% heat-inactivated fetal calf serum (Gibco, Grand Island, NY, USA) at 37 °C under a humidified atmosphere with 5% CO_2_. The medium was renewed twice a week.

#### 3.9.2. Macrophage Differentiation and Quantitative Cytokines Trial

The polysaccharide components obtained though freeze-drying were prepared with PBS solution and centrifuged for 30 min. The supernatants were diluted to 0.5, 2, and 5 mg/mL (final concentration of 50, 200, and 500 μg/mL). THP-1 cells were differentiated by phorbol 12-myristate 13-acetate (PMA, Sigma-Aldrich) to form a mature macrophage-like state. The medium was removed after treatment with PMA for 48 h. The fresh medium (180 μL) and serial concentrations of samples (20 μL) were added into each well of a 96-well plate. PBS and LPS (100 ng/mL) were served as negative and positive controls, respectively. After being co-cultured for 48 h, the cell supernatants were collected by centrifugation at 400× *g* for 3 min. The levels (pg/mL) of cytokines in culture supernatant were determined synchronously by an ELISA kit according to the manufacturer’s instructions. The cell conditioned culture solution was dissolved at room temperature, and the same volume of cell conditioned culture solution was obtained according to the instructions of the ELISA kit. The cells were added to the ELISA 96-well plate coated with human IL-6, IL-10, IL-β, and TNF-α McAb, respectively. A double antibody sandwich assay was used to detect the expression of two cytokines. After the reaction was terminated, the absorbance of 450 nm was measured. According to the standard curve, the OD values of each group of samples were converted to the concentrations of human IL-6, IL-10, IL-β, and TNF-α in the conditioned medium.

### 3.10. Statistical Analysis

All data were expressed as mean ± SD. Differences between the groups were analyzed through a one-way analysis of variance (ANOVA) followed by Newman-Keuls post hoc test. *p* values less than 0.05 (*p* < 0.05) were considered statistically significant. Interventionary studies involving animals or humans, and other studies requiring ethical approval, must list the authority that provided approval and the corresponding ethical approval code.

## 4. Conclusions

*H. erinaceus* is a kind of edible and medicinal mushroom known for its delicious role and various biological activities. The results of this study showed the fruiting body of *H. erinaceus* cultivated by the 321 strain induced by ARTP mutagenesis had better agronomic characteristics and higher polysaccharide content than the original one. Meanwhile, the physical and chemical properties, structural characteristics, and immune activities of increasing grade ethanol precipitated polysaccharides from the fruiting bodies of ARTP mutagenesis *H. erinaceus* were significantly different from those of the original ones. The effects of ARTP mutation on the physicochemical properties and bioactivities of polysaccharides from the fruiting bodies of *H. erinaceus* were investigated, which clarified that the enhancement of immune activity on polysaccharides from the ARTP mutagenesis was related to the changes of its structure characteristics, including polysaccharide distribution with large molecular weight, proportion of monosaccharide composition, and polysaccharide content. This would provide a theoretical reference for the application of ARTP mutagenesis in the quality breeding of *H. erinaceus* and it may also lay a foundation for better exploitation and utilization of *H. erinaceus* resources and further exploration on the mechanism of polysaccharide synthesis.

## Figures and Tables

**Table 1:** Comparative analysis of agronomic characteristics of *H. erinaceus*.

Sample	Shape	Fleshy Quality	Spiny Length/cm	Yield/g	Polysaccharide content (%)
0605	Round	Tight relatively	1.31 ± 1.09	113 ± 11.16 ^a^	5.16 ± 0.25 ^c^
321	Large and round	Tight	1.83 ± 0.22	138 ± 5.37 ^b^	6.03 ± 0.09 ^d^

Values shown of the samples (*n* = 100); ^a–d^ Values in the same column with different superscript letters are significantly different (*p* < 0.05).

**Table 2:** Molecular weight distribution of 20%, 50%, and 70% ethanol precipitated polysaccharides from original *H. erinaceus* (H1P) and ARTP mutagenic *H. erinaceus* (H2P).

Fraction	Peak1				Peak2			
	Mw (Da)	Mn (Da)	Mw/Mn	Ratio (%)	Mw (Da)	Mn (Da)	Mw/Mn	Ratio (%)
H1P20	6.30 × 10^6^	5.84 × 10^6^	1.16	51.7	2.33 × 10^5^	1.87 × 10^5^	1.87	48.3
H2P20	2.18 × 107	2.04 × 10^7^	1.07	78.8	2.80 × 10^5^	2.49 × 10^5^	1.50	21.2
H1P50	2.27 × 10^4^	2.22 × 10^4^	1.11	100	-	-	-	-
H2P50	6.52 × 10^4^	4.27 × 10^4^	1.47	100	-	-	-	-
H1P70	1.65 × 10^4^	1.33 × 10^4^	1.23	15.5	3.97 × 10^4^	3.08 × 10^4^	1.28	84.5
H2P70	9.78 × 10^4^	6.96 × 10^4^	1.40	28.1	3.77 × 10^4^	2.50 × 10^4^	1.50	71.9

H1P20 (H1P50, H1P70): 20% (50%, 70%) ethanol precipitated polysaccharides from the original *H. erinaceus*; H2P20 (H2P50, H2P70): 20% (50%, 70%) ethanol precipitated polysaccharides from the ARTP mutagenic *H. erinaceus*; Mw: molecular weight; Mn: number-average molecular weight; “-”: not detected or very little content.

**Table 3:** Monosaccharide composition of ethanol precipitated polysaccharides.

Sample	Fuc	Ara	GlcN	Gal	Glu	Xyl	Man	Fru
H1P20	1.89	1.02	0.86	0.73	3.91	0.18	1.00	-
H2P20	2.00	0.63	0.41	1.30	4.98	0.75	1.00	0.56
H1P50	1.29	-	0.19	1.74	2.45	-	1.00	-
H2P50	7.12	0.16	0.29	3.72	4.35	0.21	1.00	-
H1P70	0.81	-	-	2.46	2.82	-	1.00	-
H2P70	3.71	-	0.21	2.33	9.99	-	1.00	-

Fuc: fucose; Ara: arabinose; GlcN: Glucosamine; Gal: galactose; Glu: glucose; Xyl: xylose; Man: mannose; Fru: fructose; H1P20 (H1P50, H1P70): 20% (50%, 70%) ethanol precipitated polysaccharides from the original *H. erinaceus*; H2P20 (H2P50, H2P70): 20% (50%, 70%) ethanol precipitated polysaccharides from the ARTP mutagenic *H. erinaceus*; “-”: not detected.
